# Analysis of factors associated with extended recovery time after colonoscopy

**DOI:** 10.1371/journal.pone.0199246

**Published:** 2018-06-21

**Authors:** Patrick C. Eschenfeldt, Uri Kartoun, Curtis R. Heberle, Chung Yin Kong, Norman S. Nishioka, Kenney Ng, Sagar Kamarthi, Chin Hur

**Affiliations:** 1 Institute for Technology Assessment, Massachusetts General Hospital, Boston, MA, United States of America; 2 Gastrointestinal Unit, Massachusetts General Hospital, Boston, MA, United States of America; 3 Harvard Medical School, Boston, MA, United States of America; 4 Center for Computational Health, IBM Research, Cambridge, MA, United States of America; 5 Northeastern University College of Engineering, Boston, MA, United States of America; University Hospital Llandough, UNITED KINGDOM

## Abstract

**Background & aims:**

A common limiting factor in the throughput of gastrointestinal endoscopy units is the availability of space for patients to recover post-procedure. This study sought to identify predictors of abnormally long recovery time after colonoscopy performed with procedural sedation. In clinical research, this type of study would be performed using only one regression modeling approach. A goal of this study was to apply various “machine learning” techniques to see if better prediction could be achieved.

**Methods:**

Procedural data for 31,442 colonoscopies performed on 29,905 adult patients at Massachusetts General Hospital from 2011 to 2015 were analyzed to identify potential predictors of long recovery times. These data included the identities of hospital personnel, and the initial statistical analysis focused on the impact of these personnel on recovery time via multivariate logistic regression. Secondary analyses included more information on patient vitals both to identify secondary predictors and to predict long recoveries using more complex techniques.

**Results:**

In univariate analysis, the endoscopist, procedure room nurse, recovery room nurse, and surgical technician all showed a statistically significant relationship to long recovery times, with p-value below 0.0001 in all cases. In the multivariate logistic regression, the most significant predictor of a long recovery time was the identity of the recovery room nurse, with the endoscopist also showing a statistically significant relationship with a weaker effect. Complex techniques led to a negligible improvement over simple techniques in prediction of long recovery periods.

**Conclusion:**

The hospital personnel involved in performing a colonoscopy show a strong association with the likelihood of a patient spending an abnormally long time recovering from the procedure, with the most pronounced effect for the nurse in the recovery room. The application of more advanced approaches to improve prediction in this clinical data set only yielded modest improvements.

## Introduction

In a gastrointestinal endoscopy unit, a common limiting factor on the number of procedures that can be performed is the availability of space for patients to recover post-procedure. Although most patients recover and are discharged in under 70 minutes, in some cases patients require 100 minutes or more of recovery room time. It is, therefore, of significant interest to understand the factors that contribute to abnormally long recovery times. Possible explanations could include the difficulty of the endoscopy, the amount of sedation used, the duration of the procedure, and the staff involved in the procedure component or recovery portion. Such an understanding could inform procedure planning and point towards interventions which may improve both the patient experience and endoscopic throughput. The aim of this study was to identify predictors of extended recovery time after colonoscopy performed with procedural sedation. The impact of colonoscopy techniques on recovery time has been examined in several randomized controlled trials, particularly comparing different sedation methods [1–3], and patient characteristics impact the overall patient time commitment for a colonoscopy [4], but the present authors are not aware of previous studies linking colonoscopy recovery time to a wide variety of patient and procedure characteristics. Because studies have identified clinician characteristics and behaviors as significant predictors of patient outcomes in other situations [5–7], we focus on the identity of the personnel involved in the procedure as a major focus of our exploration.

Along with the identification of factors impacting recovery time, there may also be some utility in designing a system to predict at the end of a colonoscopy whether a particular patient will require an abnormally long recovery time. Such a system could be integrated into a broader operations management scheme for the endoscopy unit to maximize the efficient use of resources. The task of predicting an outcome from data can be approached in a classical statistical way or via a variety of more modern so-called “machine learning” methods. Previous research comparing simpler traditional and more complex machine learning approaches in medical applications has shown mixed results, with simple methods often but not always performing as well as more complex techniques [8–12]. The aim of this study was to compare various techniques for the prediction of long colonoscopy recovery times.

## Materials and methods

### Data collection

From 2011 to 2015, the MetaVision system was used to record both procedural and patient data about endoscopies performed at the Massachusetts General Hospital (MGH). The MetaVision system [13] is an anesthesia information management system that automatically records and documents patient vitals from various connected devices and provides an interface for physicians and nurses to enter additional data in real time, including administration of drugs and qualitative assessments of patient state. The system has been used to facilitate a variety of research projects and quality improvement efforts [14–18]. Recorded patient data included limited demographic information and vital signs during the procedure. Other information included the names of the physician, nurse, and hospital staff involved in the procedure and the dosages of drugs given to the patient during the procedure. The precise times of events such as the start and end of the procedure, when drugs were administered, and when the patient was discharged were also recorded electronically. The institutional review board of Partners Healthcare approved the use of these data (protocol number 2016P002243), which were collected as part of standard clinical care.

### Study population

During the observation window of October 3, 2011, to June 30, 2015, doctors performed 31,852 colonoscopies on adult patients at MGH, and the procedures were recorded in the MetaVision system. The recovery time was defined as the time between the recorded end of the procedure and the time the patient was discharged. Procedures for which an accurate recovery time could not be calculated based on the recorded data (*n* = 274) and for which the patient was not discharged on the same day that the procedure ended (*n* = 20) were excluded from analysis. Because standard practice at MGH during the observation window indicated the use of propofol only for colonoscopies performed under general anesthesia, procedures in which propofol was administered (*n* = 116) were also excluded. The final population analyzed was 31,442 colonoscopies involving 29,905 patients.

### Statistical analysis

To determine predictors for unusually long recovery times, the distribution of recovery time in the study population was analyzed. The 80th percentile of recovery time was identified and used to define a long recovery. This definition of a long recovery was motivated by the operational impact of abnormally long recovery times on endoscopy units.

Initial potential predictors were selected to allow investigation of the impact of hospital personnel on the likelihood of a long recovery time. These covariates were the endoscopist who performed the procedure, the nurse in the procedure room, the surgical technician assisting on the procedure, and the nurse in the recovery room. In cases where multiple personnel filled a given role, the one entered into the primary field in the MetaVision database was selected. To determine if any differences between personnel was primarily shown by individual patterns in drug administration, total dosages throughout the procedure of fentanyl, meperidine, midazolam, diphenhydramine, and ondansetron were computed for each procedure. Patient gender, age, and American Society of Anesthesiologists (ASA) classification were included as potential confounders along with the year of the procedure.

For hospital personnel, individuals who did not participate in enough procedures (fewer than 50 for endoscopists and nurses, fewer than 20 for technicians) were grouped together as “other.” Additionally, gastroenterology fellows listed as primary endoscopists were grouped together, with a separate group for fellows who became full-time staff during the observation period. After these consolidations, there were 35 endoscopists, 74 procedure room nurses, 15 technicians, and 97 recovery room nurses. To explore the relationship between each of these groups and recovery time, the mean and median recovery time for each individual were computed, and one-way analysis of variance (ANOVA) was performed to examine the significance of the relationship.

Univariate analyses of all selected variables were performed using the Mann-Whitney test for continuous variables and the Pearson *χ*^2^ test for categorical variables. Statistically significant variables with *p* < 0.05 were then entered into a multivariate logistic regression model. To account for the large number of possible values of personnel variables, individual personnel were sorted by mean recovery time and split into quintiles. These quintiles were entered as categorical variables in the multivariate model. All statistical analysis was performed using R version 3.4 [19].

### Secondary factor identification

To identify factors associated with long recovery beyond those discussed above, a larger set of covariates was identified from the MetaVision database. Analysis of these variables was performed independently on subsets of the database split by the quintile of the recovery room nurse. Potential predictors were selected from all variables available in the MetaVision database by a preliminary scan for a significant relationship with long recovery times followed by stepwise logistic regression. Selected covariates included a variety of quantitative and qualitative measurements of the patient’s state during the procedure. Univariate analyses were performed using the procedure described above with statistically significant variables entered into a multivariate logistic regression. The results of these regression models for each quintile of recovery room nurse were compared to identify common trends.

### Supplemental prediction models

In addition to the descriptive analysis, several more complex models were built with a specific goal of predicting whether a procedure would result in a long recovery time, even if the model was potentially less interpretable or did not directly identify important predictors. Because of the operational importance of avoiding abnormally long recovery times rather than analyzing variation in shorter times, classification models were built to predict such long times, rather than using regression models to directly predict recovery time itself. These models were trained on a randomly selected subset of 23,582 (75%) of the procedures and tested on the remaining 7,860 (25%). Note that for the predictive models, the personnel quintiles were defined based only on the training set. Models were evaluated by the area under the receiver operating characteristic (ROC) curve (AUC). One of these models was the multivariate logistic regression described above. Other models incorporated more variables from the MetaVision system, including the preliminary findings of the exam, the patient’s heart rate, diastolic and mean blood pressure, and the patient’s observed pain level and level of consciousness at the end of the procedure. These variables were selected from the set of all variables available in the MetaVision database by a two step process: first univariate statistical tests were performed for each variable and those without a significant relationship with long recovery times were discarded, then remaining variables were added one at a time into multivariate logistic regression models with the variable selected at each step determined by the model that achieved the highest AUC on the training set. The final set of variables was chosen to be the smallest set from this sequence for which adding the next variable resulted in a minimal increase in AUC.

This variable set was used to train a variety of models, described below. The selection of models chosen reflects a range of so-called “machine learning” methods with accessible implementations in R. The settings used for these methods generally followed implementation defaults except where intuition or preliminary experimentation on the training data suggested that tuning of parameters would have a significant impact on the performance of the model. For most such parameters cross-validation was used to select optimal values, with cross-validation settings selected to balance model performance and computation time.

#### Logistic regression

Along with the multivariate logistic regression on the original variable set used in the descriptive analysis, a standard multivariate logistic regression was performed with the expanded variable set used for other prediction models.

#### Decision trees

Three decision tree models were fit. First, using the R package tree [20], a model was fit using the default settings, splitting based on deviance. Then, using the R package rpart [21], a model was fit splitting on Gini impurity with the relative loss for a false negative selected to maximize AUC by 10-fold cross-validation on the training set. This loss weighting was performed to compensate for the imbalanced classes, and the value was selected from the set {0.5, 1, 5, 10, 50}. Cross-validation was performed with the R package caret [22]. Finally, a conditional inference tree model was fit using the default settings for the function ctree in the R package party [23].

#### Random forest

Using the R package randomForest [24], a random forest model was fit with 2000 trees and otherwise using default settings. In particular the minimum size for terminal nodes in each tree was one.

#### Lasso regression

Lasso regression was performed using the built-in function cv.glmnet from the R package glmnet [25], using 10-fold cross-validation to select the regularization parameter from the candidate sequence {*e*^−10+0.015*i*^ ∣ *i* = 0, 1, … , 1000}. Adaptive lasso regression [26] was performed by using cv.glmnet to perform ridge regression to select weights using default cross-validation settings, with constant *γ* = 1. These weights are then used in another call to cv.glmnet to compute the adaptive lasso model, where 10-fold cross-validation selected the regularization parameter from candidate sequence {*e*^−9+0.019*i*^ ∣ *i* = 0, 1, … , 1000}. For both lasso and adaptive lasso, model coefficients were extracted, with confidence intervals generated by bootstrapping, for qualitative assessment of variable importance. All covariates were standardized to mean zero and standard deviation one before fitting the model.

#### Stepwise logistic regression

Stepwise logistic models were built using the core R function step, fitting between an empty model and a model including all variables available to other models. Forward, backward, and bidirectional selection were performed, with the latter starting with the empty model.

#### Neural network

A fully-connected single-layer feed-forward neural network was built using the R package nnet [27]. This package uses a sigmoid activation function and performs the training optimization via the “BFGS” quasi-Newton method as implemented in the core R function optim. Twice-repeated 5-fold cross-validation maximizing AUC via the R package caret was used to select the number of hidden nodes and decay parameter. The latter parameter penalizes large weights to avoid overfitting the model. The cross-validation selected the number of nodes from {10, 15, 30, 1.5*m*, 2*m*} where *m* is the number of variables in the model (including dummy variables replacing categorical variables) and the decay parameter from {0, 0.1, 5, 10, 100}. To allow the algorithm to run for all tested parameter combinations, the maximum allowed iterations and number of weights were increased to 20,000 and 3,000, respectively. All covariates were scaled and centered to be mean zero and standard deviation one before training the model.

#### Support vector machine

A support vector machine with a radial basis kernel function was fit using the R package e1071 [28]. Twice-repeated 10-fold cross-validation minimizing classification error was performed using the e1071 function tune to select the kernel parameter *γ* from the set {0.001, 1, 10} and cost of constraint violation from the set {10^−2^, 10^1.5^, 10^5^}. Decision values were used to compute the AUC.

## Results

**Fig 1:**
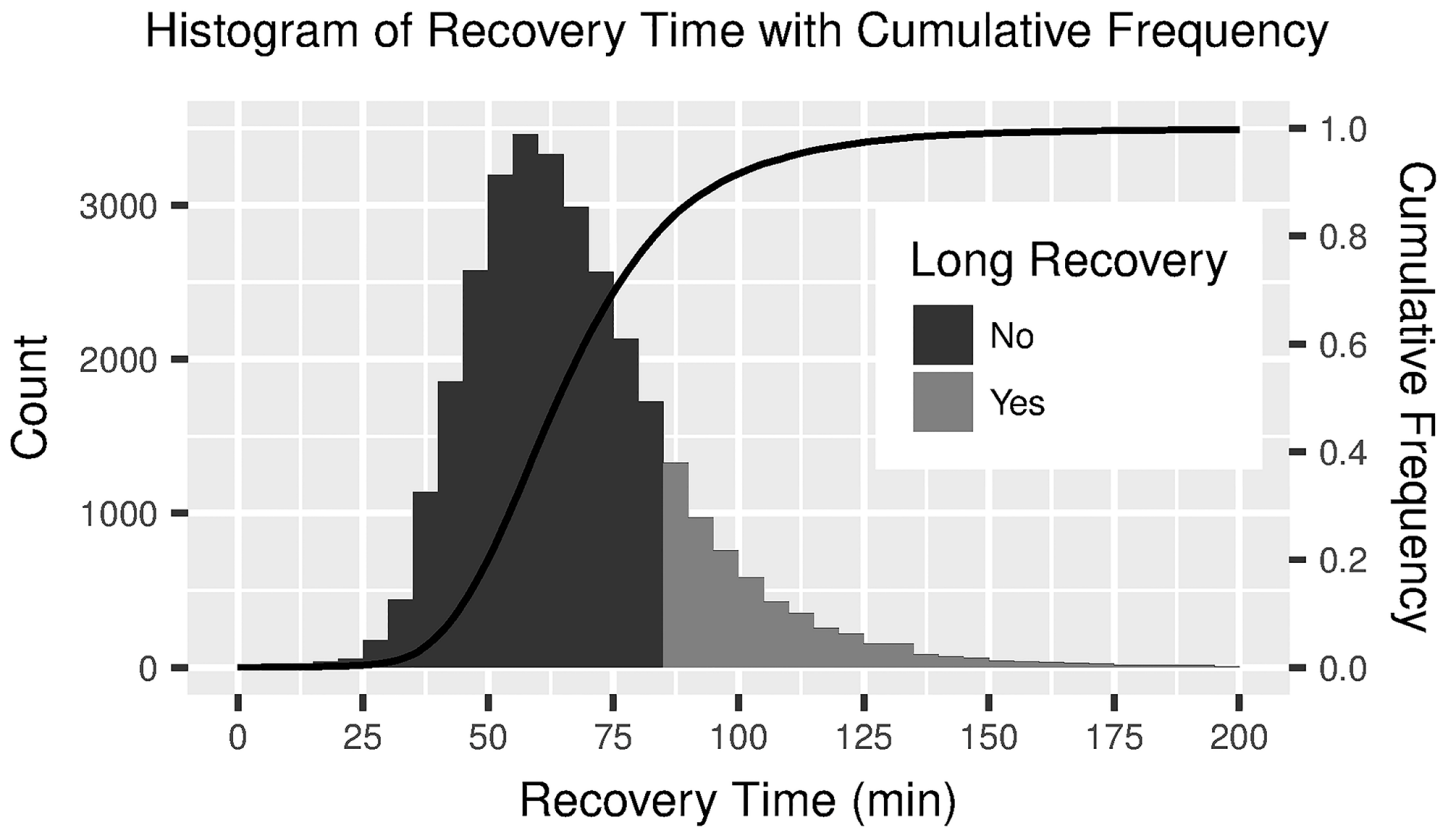
Histogram of recovery time with cumulative frequency. Distribution of recovery time among studied population, showing definition of long recovery. For readability, the 71 (1.4%) procedures with recovery time above 200 minutes are excluded from the figure.

For the 31,442 procedures analyzed in this study, median recovery time was 65 minutes (interquartile range (IQR) 53–80). A long recovery was defined as longer than 85 minutes; 5,718 (18.2%) procedures resulted in a long recovery time. Fig 1 presents a histogram of recovery time.

**Fig 2:**
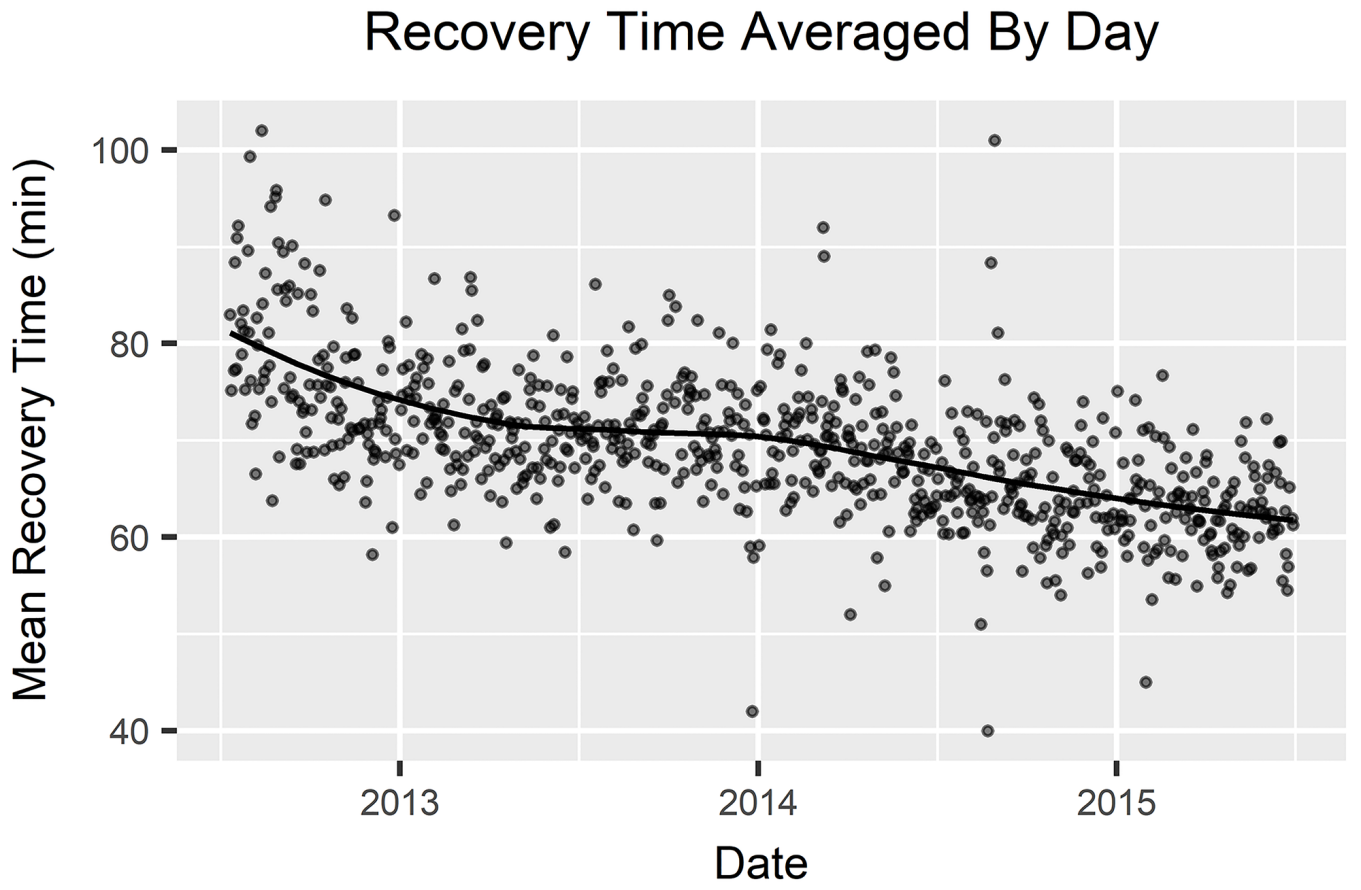
Recovery time averaged by day. Daily mean recovery time over the study period with a local regression (LOESS) fitted curve.

**Fig 3:**
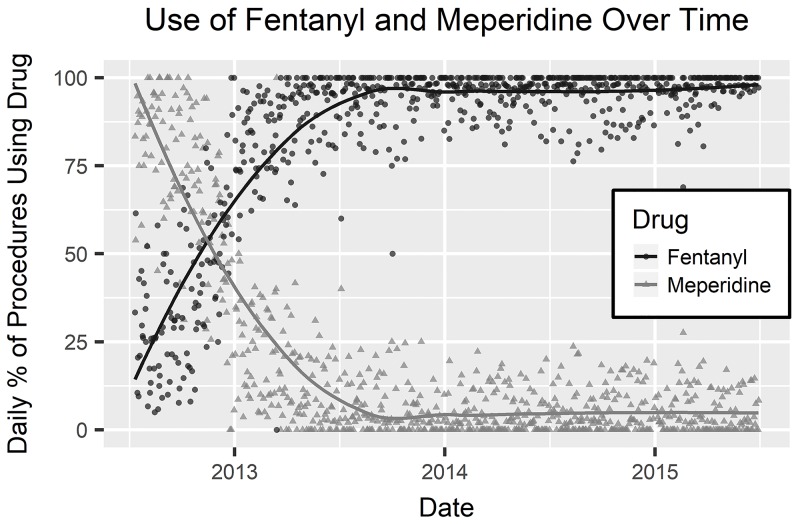
Use of fentanyl and meperidine over time. Daily percentage of procedures using fentanyl and meperidine over time, with LOESS fitted curves.

Women were more likely than men to have a long recovery period. There was no difference in the median ages of the patients. Patients with ASA classification 2 were somewhat less likely to have a long recovery time than all other classes or those with no classification entered into the database. Procedures with a long recovery time were more likely to have been performed in the first two years of the study period. This matches an observed trend over time toward shorter recovery time; Fig 2 shows this general change over the study period. All four observed hospital personnel variables showed a significant relationship to whether recovery time was long or not. Total received dosage of all drugs also showed a significant relationship with long recovery times. This relationship appears to be driven by whether a patient received a particular drug more than by the dosage received. Patients with long recovery times were less likely to have received any dosage of fentanyl, but more likely to have received each of the other drugs in this study. Median dosages of drugs, when used at all, were similar between long recovery procedures and others. Note that drug usage patterns changed over the course of the study period; as shown in Fig 3, the percentage of procedures using meperidine decreased over the first year and the percentage using fentanyl increased concurrently reflecting a new recommended policy that was implemented during the study period. Table 1 summarizes univariate analyses.

**Table 1:** Procedure characteristics by recovery time.

Variable	Recovery Time		p value*
	≤ 85 min n = 25,724	> 85 min n = 5,718	
Patient Demographics			
Female (%)	48.38	61.75	<0.0001
Age (median (IQR))	59 (51, 67)	59 (50, 67)	0.0194
ASA Class			0.0032
Class 1 (%)	24.52	26.27	
Class 2 (%)	73.40	71.28	
Class 3/4/Unknown (%)	2.08	2.45	
Year			<0.0001
2012 (%)	9.06	15.76	
2013 (%)	34.73	41.27	
2014 (%)	37.95	32.51	
2015 (%)	18.26	10.46	
Personnel^†^			
Endoscopist	-	-	<0.0001
Procedure RN	-	-	<0.0001
Recovery RN	-	-	<0.0001
Technician	-	-	<0.0001
Drugs			
Diphenhydramine (mg)^‡^	3.3/50 (25, 50)	6.3/50 (25, 50)	<0.0001
Fentanyl (mg)^‡^	89.8/0.10 (0.10, 0.15)	83.6/0.13 (0.10, 0.15)	0.0001
Meperidine (mg)^‡^	11.7/75 (50, 100)	21.8/75 (50, 100)	<0.0001
Midazolam (mg)^‡^	98.8/4.5 (4.0, 5.5)	99.8/5.0 (4.0, 6.0)	<0.0001
Ondansetron (mg)^‡^	3.4/4.0 (4.0, 4.0)	7.7/4.0 (4.0, 4.0)	<0.0001

* p values from Mann-Whitney test for continuous variables and Pearson chi-square test for categorical variables.

^†^ Individual percentages omitted due to the large number of individuals for each category.

^‡^ Percentage of procedures with any use of drug / median (IQR) among procedures with nonzero use.

**Fig 4:**
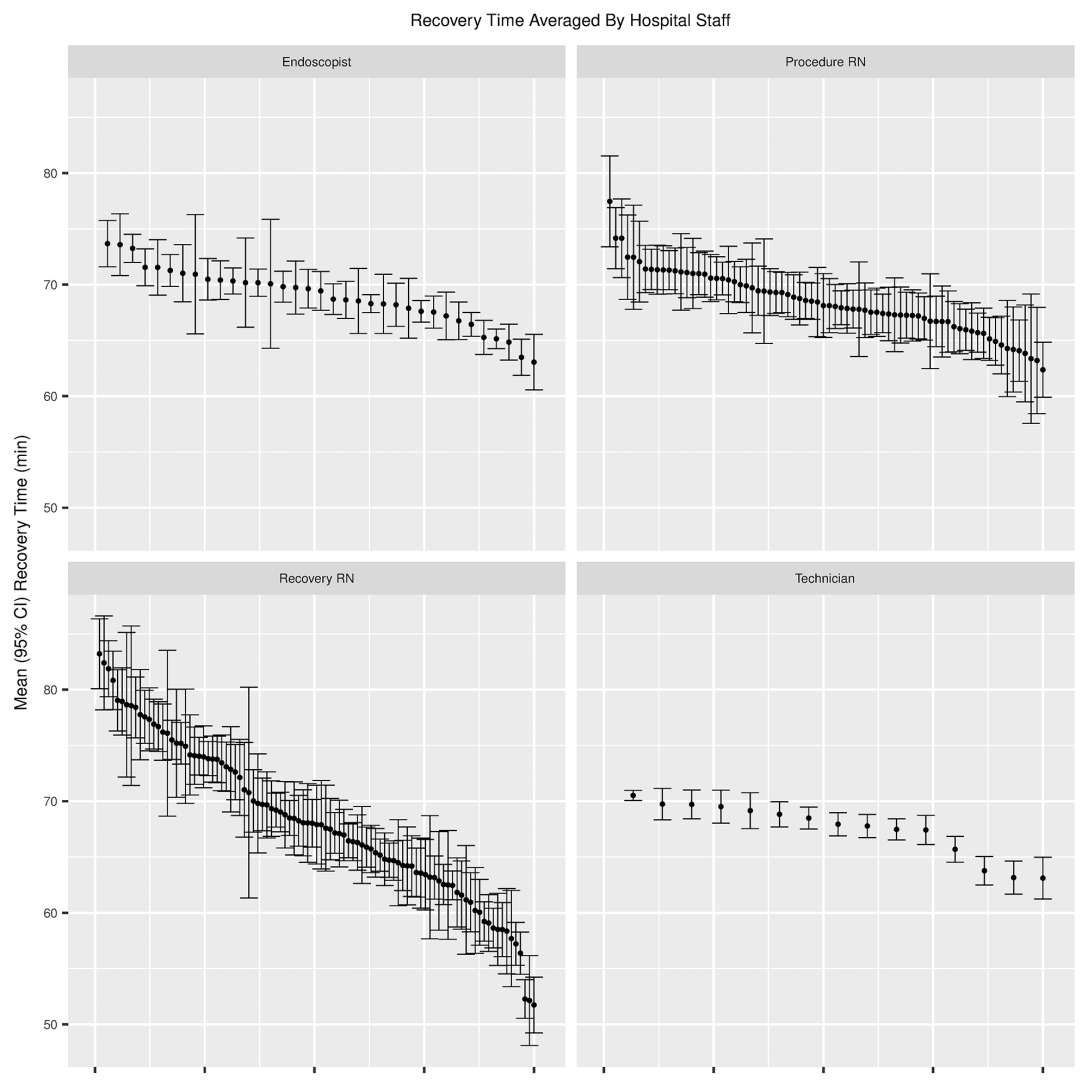
Recovery time averaged by hospital staff. Mean recovery time by hospital personnel with 95% confidence interval. Each point represents one individual or the aggregated data of individuals involved in a small number of procedures, as described in the methods section.

The mean recovery time for individual endoscopists varied by 10 minutes from shortest to longest; 15 minutes for procedure room nurses, 8 minutes for technicians, and 31 minutes for recovery room nurses. The equivalent differences for median recovery time were 11 minutes for endoscopists, 15 minutes for procedure room nurses, 8 minutes for technicians, and 32 minutes for recovery room nurses. Fig 4 summarizes mean recovery time by personnel and similar figures for median recovery time and fraction of procedures with long recovery times are available in S1 and S2 Figs. ANOVA for each of the personnel variables shows that the means vary significantly by individual, with p-value less than 0.0001 in each case.

In the multivariate regression model, the first quintile of each personnel variable, that is the quintile of individuals with the shortest average recovery time, was used as the baseline. For other categorical variables, the baseline was female gender, ASA class 1, and procedure year 2012. In this regression analysis, gender, age, years of service 2014 and 2015 were significant at a p-value of 0.0001 level, as were meperidine, diphenydramine, and ondansetron. All quintiles of endoscopist and recovery nurse were significant at a p-value of 0.0001 level. The odds ratios for all quintiles of recovery nurse are larger than for any quintile of endoscopist (or other personnel), and the odds ratios for the top two quintiles of recovery nurse are more than twice those associated with any other personnel. Drug dosages all have small odds ratios relative to recovery nurse and endoscopist. Table 2 summarizes multivariate regression results.

**Table 2:** Multivariate regression results.

Variable	Odds Ratio of Long Recovery	Lower (95% CI)	Upper (95% CI)	p value
Gender: Male vs. Female	0.63	0.59	0.67	<0.0001
Age*	1.08	1.05	1.11	<0.0001
ASA Class:				
2 vs. 1	0.98	0.91	1.05	0.5878
3/4/Unknown vs. 1	1.44	1.17	1.77	0.0006
Year:				
2013 vs. 2012	0.83	0.74	0.92	0.0007
2014 vs. 2012	0.66	0.59	0.74	<0.0001
2015 vs. 2012	0.49	0.43	0.57	<0.0001
Endoscopist Quintile:				
2^nd^ vs. 1^st^	1.28	1.17	1.40	<0.0001
3^rd^ vs. 1^st^	1.28	1.16	1.42	<0.0001
4^th^ vs. 1^st^	1.42	1.29	1.56	<0.0001
5^th^ vs. 1^st^	1.56	1.41	1.72	<0.0001
Procedure RN Quintile:				
2^nd^ vs. 1^st^	1.11	0.99	1.24	0.0781
3^rd^ vs. 1^st^	1.05	0.94	1.17	0.4294
4^th^ vs. 1^st^	1.15	1.04	1.28	0.0085
5^th^ vs. 1^st^	1.23	1.10	1.38	0.0003
Recovery RN Quintile:				
2^nd^ vs. 1^st^	1.66	1.47	1.89	<0.0001
3^rd^ vs. 1^st^	2.13	1.89	2.41	<0.0001
4^th^ vs. 1^st^	3.13	2.79	3.53	<0.0001
5^th^ vs. 1^st^	5.22	4.64	5.87	<0.0001
Technician Quintile:				
2^nd^ vs. 1^st^	1.02	0.88	1.19	0.7537
3^rd^ vs. 1^st^	1.11	0.95	1.30	0.1926
4^th^ vs. 1^st^	1.11	0.95	1.31	0.1778
5^th^ vs. 1^st^	1.08	0.94	1.25	0.2901
Diphenhydramine*	1.15	1.11	1.19	<0.0001
Fentanyl*	1.16	1.04	1.29	0.0061
Meperidine*	1.07	1.05	1.09	<0.0001
Midazolam*	1.07	1.03	1.11	0.0006
Ondansetron*	1.18	1.14	1.21	<0.0001

* Odds ratio per 10 year increase in age and increase in dosage of drugs: 10 mg for diphenhydramine and meperidine, 1 mg for midazolam and ondansetron, and 0.1 mg for fentanyl.

For each quintile of recovery room nurse, variables were selected from a broad set of quantitative and qualitative information recorded during the procedure. Univariate analysis selected 23 covariates for the first quintile, that is the 20% of nurses with the shortest average recovery time. For the second quintile, 30 variables were selected, 18 for the third quintile, 24 for the fourth, and 24 for the fifth quintile. In the multivariate regression models, 4 covariates had a p-value below 0.05 for all quintiles: patient consciousness at the end of the procedure, dosage of diphenhydramine and ondansetron, and endoscopist in the fifth quintile (with the first as the reference class). Of these covariates, patient consciousness displayed the greatest effect, with drowsy patients associated with approximately 1.5–2 times the number of long recoveries of alert patients. Some covariates were statistically significant predictors with a large effect for a subset of recovery nurse quintiles; patient pain level at the end of the procedure rated as anything other than no pain had odds ratio 6.92 (2.90, 15.58) for the first quintile, 6.54 (3.23, 13.31) for the second quintile, 3.30 (1.61, 6.56) for the third quintile, and 2.01 (1.04, 3.77) for the fourth quintile, but was not statistically significant for the fifth quintile with odds ratio 1.53 (0.83, 2.77) and p-value 0.16. All odds ratios for the multivariate logistic regressions on each nurse subset set can be found in S3 Table.

### Predictive model comparison

Among all the predictive models, the neural network performed the best, achieving an AUC of 0.723 (0.710, 0.737) on the test set, compared to 0.722 (0.708, 0.736) for the multivariate logistic regression on the expanded variable set and 0.697 (0.682, 0.712) for the multivariate logistic regression on the original variable set described in Table 2. The single layer neural network chosen by cross-validation had 66 nodes in the hidden layer and regularization decay parameter equal to 10. Other models had comparable or worse performance on the test set. All model results are summarized in Table 3.

**Table 3:** Predictive model results.

Method	Training set AUC (95% CI) n = 23,582	Test set AUC (95% CI) n = 7,860
Neural Net	0.747 (0.739, 0.755)	0.723 (0.710, 0.738)
Logistic Regression (expanded)	0.734 (0.725, 0.742)	0.722 (0.708, 0.736)
Lasso	0.728 (0.720, 0.736)	0.718 (0.704, 0.733)
Adaptive Lasso	0.724 (0.716, 0.732)	0.716 (0.702, 0.730)
Random Forest	1.000 (1.000, 1.000)	0.715 (0.701, 0.730)
Logistic Regression	0.703 (0.695, 0.711)	0.697 (0.682, 0.712)
Decision Tree (ctree)*	0.711 (0.704, 0.720)	0.676 (0.661, 0.691)
Decision Tree (rpart)*	0.642 (0.634, 0.650)	0.640 (0.626, 0.654)
Support Vector Machine	0.791 (0.783, 0.799)	0.637 (0.620, 0.653)
Decision Tree (tree)*	0.610 (0.602, 0.618)	0.617 (0.603, 0.630)

* tree and rpart represent different implentations of standard decision trees, ctree is a conditional inference tree.

Table 3 includes performance on the training set as well as the test set; the former is included because a large gap between training and test performance can be indicative of overfitting for some models. For most models, the observed gap is relatively small, with the exception of the support vector machine and the random forest. Note, however, that perfect accuracy on the training set is expected for the random forest model because each tree is grown to a maximal size that will have only one training example per terminal node and thus will correctly classify any example which was selected by the bootstrapping for that tree. As more than half of the bootstrap samples will contain any given training example, all training examples will be properly classified by more than half of the trees and therefore properly classified by the forest. The support vector machine may be overfit to the training data because it was the most computationally intensive of our models and thus cross-validation selected from only a small subset of parameter values.

Among the variables added for the predictive models, the patient’s pain level at the end of the procedure has the largest effect in the logistic regression, with any pain level of at least 1 out of 10 having an odds ratio of 2.86 (2.01, 4.07) versus a pain level of zero in the trained logistic regression. Note that recovery room nurse remains the largest effect overall, with the fourth quintile vs. the first having odds ratio of 3.07 (2.68, 3.53) and the fifth vs. the first having odds ratio 5.41 (4.72, 6.22). Patient consciousness at the end of the procedure also has a strong association, with drowsy patients having an odds ratio of 1.71 (1.56, 1.88) versus alert patients and patients requiring stronger stimulation to arouse having an odds ratio of 2.22 (1.75, 2.81). Patient heart rate and blood pressure also show a statistically significant association with extended recovery times. All odds ratios for the multivariate logistic regression on the expanded variable set can be found in S2 Table. The lasso and adaptive lasso regressions led to similar qualitative results, with recovery room nurse still having the largest effect and pain and consciousness variables also showing a strong association.

## Discussion

This retrospective cohort study of colonoscopies performed at MGH has identified several factors that are associated with undesirable long recovery times. One factor was the date of the procedure, as there was a general trend over time for recovery times to become shorter and long recoveries to become rarer. We believe this change to be at least in part caused by specific efforts of the endoscopy unit at MGH to increase throughput. These efforts included the director of endoscopy meeting with the endoscopy unit staff to highlight these goals a total of three times as well as the recommended change from meperidine to fentanyl for the majority of cases.

We also observed that women were significantly more likely than men to experience a long recovery time. While we were unable to perform an extensive evaluation of this effect, we do observe that gender was a significant predictor in the multivariate logistic regression on the expanded variable set described in S2 Table. This finding could be explored in future analyses.

Procedure characteristics associated with long recoveries included some non- intervenable patient characteristics such as gender and age, but some potentially actionable factors were also identified even after accounting for basic patient characteristics and the previously mentioned general trend over time. The most directly modifiable predictors with a statistically significant association with long recovery times were drug dosages, with patients receiving larger dosages of all drugs being consistently slightly more likely to require a long recovery time. This effect is observed both in the primary model and in the expanded model which adjusts for patient consciousness and pain at the end of the procedure, suggesting that the increased likelihood of long recovery from increased drug dosage is not entirely caused by the effect of drug dosage on patient state. This suggests that decreasing drug dosages may lead to a small decrease in long recovery times, but this analysis cannot and does not fully account for the impact of such dosage changes on patients, including effects which may counter the recovery time advantages of lower dosages.

While the drug dosage effect was statistically significant, the identity of the endoscopist performing the procedure and the nurse in the recovery room both showed a much larger effect. Recovery room nurses with the longest average recovery times were associated with 5 times as many long recovery procedures as those with the shortest average recovery times. The drastically greater variation between recovery room nurses than between other personnel suggests that interventions targeted at recovery room nurses may be more effective than those focusing on endoscopists’ behavior. The data used in this study are not adequate for informing most such interventions, as they do not capture nurse behavior in the recovery room or any nurse characteristics.

While the subset analysis performed by grouping procedures together by quintiles of recovery room nurse also does not provide insight into nurse behavior, it may demonstrate some interactions between nurses and other factors. For example, the effect of the most extreme endoscopists is relatively stable between nurse quintiles, suggesting this effect acts independently from the nurse effect. One effect that behaves differently is patient pain, which is statistically significant for the lower four quintiles of recovery room nurses but not for the fifth quintile. One possible explanation for this effect is that most nurses respond to patients who have been in pain by allowing or encouraging a longer recovery time but those in the fifth quintile are generally so much more likely to oversee longer recovery times that the presence or absence of pain doesn’t have an impact on their behavior. Some sort of direct observation or significantly more detailed data collection would likely be necessary to confirm any speculation on this question, or any similar questions about the relationship between nurse identity and long recoveries.

As we stated above, however, the dataset used in this study does contain additional procedural and patient data that may be useful for simple prediction of whether a patient leaving a completed procedure will experience a long recovery time. Our predictive models show that the most useful additional information for such predictions relates to the state of the patient at the end of the procedure; for example if they are in pain or are less than fully conscious. A prediction tool built from these data may be of some operational interest for endoscopic facilities. Our analysis applied various models to optimize prediction and demonstrated that there may be some advantage, albeit a small one, to complex “machine learning” techniques. However, using our data set, the much less computationally intensive multivariate logistic regression was essentially as effective as the neural network model. That the logistic regression and neural network models performed nearly identically when provided with the same features perhaps suggests that the relationships between those features in this dataset are well-modeled by the assumptions of logistic regression, or that the increased flexibility of the neural network does not capture any more complex relationships.

### Future work

The most immediate avenue for future work beyond this analysis is to further clarify and understand the impact of recovery room nurses on long recovery times by more directly studying nurse behavior and identity. If a sufficiently detailed data set could be found this could be another retrospective data analysis, but more likely it would involve direct observation of nurses, whether as a research study or quality improvement endeavor at an endoscopy unit. Significant efforts to eliminate long recoveries should first identify recovery room nurses associated with long and short recovery times then analyze the characteristics and behaviors of those nurses to determine more specific causes of long recoveries. If differences in behavior can be correlated with recovery times and modifying or standardizing such behaviors is deemed appropriate for patient care, many long recovery times can potentially be eliminated.

Another potential avenue for future work is to perform an analysis similar to this one focusing on patients who undergo endoscopy wholly without sedation, to see if the factors impacting their recovery time are different. Given appropriate data, similar analyses could also be performed for any procedures where recovery time is an outcome of interest.

### Conclusion

In summary, we present the results of an analysis that used both more traditional biostatistical techniques and advanced machine learning methods to identify factors associated with a long post-procedure recovery time. Our study identifies potential factors, including potentially modifiable ones, that could aid in improving endoscopy unit efficiency. Our analysis also demonstrates that, at least with our data set, applying complex techniques instead of simpler and more traditional logistic regression did not provide a significant improvement in predicting long recovery times.

## Supporting information

S1 Table Personnel quintiles by recovery time. Univariate analysis of the personnel quintile variables used in the multivariate regression.
(PDF)

S2 Table Multivariate regression results (expanded variable set). Results of multivariate logistic regression on the expanded variable set.
(PDF)

S3 Table Multivariate regression results by RN quintile. Results of multivariate logistic regression models for each quintile of recovery room nurse.
(PDF)

S1 Fig Recovery time averaged by hospital staff. Median recovery time by hospital personnel with interquartile range. Each point represents one individual or the aggregated data of individuals involved in a small number of procedures, as described in the methods section.
(PDF)

S2 Fig Percentage of long recoveries by hospital staff. Percentage of procedures with recovery time greater than 85 minutes by hospital personnel with Wilson confidence interval. Each point represents one individual or the aggregated data of individuals involved in a small number of procedures, as described in the methods section.
(PDF)
