# Supplementary material for: Analysis of factors associated with extended recovery time after colonoscopy
Source: PLoS One. 2018 Jun 21;13(6):e0199246. doi: 10.1371/journal.pone.0199246 (PMC6013091; doi:10.1371/journal.pone.0199246)
Supplement: S1 Table — Univariate analysis of the personnel quintile variables used in the multivariate regression. (PDF) [file pone.0199246.s001.pdf]

Supplementary Table 1:
Personnel Quintiles By Recovery Time

Variable	Recovery Time ≤ 85 min n = 25,724	Recovery Time > 85 min n = 5,718	p value†
Endoscopist			< 0.0001
1st Quintile	24.97	18.20	
2nd Quintile	24.24	23.42	
3rd Quintile	16.19	16.37	
4th Quintile	19.41	21.44	
5th Quintile	15.19	20.57	
Procedure RN			< 0.0001
1st Quintile	14.58	10.90	
2nd Quintile	20.49	18.66	
3rd Quintile	20.54	19.29	
4th Quintile	26.04	28.24	
5th Quintile	18.35	22.91	
Recovery RN			< 0.0001
1st Quintile	19.21	7.24	
2nd Quintile	21.28	14.20	
3rd Quintile	22.14	18.82	
4th Quintile	21.68	27.46	
5th Quintile	15.69	32.28	
Technician			< 0.0001
1st Quintile	8.44	5.33	
2nd Quintile	18.40	16.46	
3rd Quintile	12.81	12.23	
4th Quintile	12.57	13.20	
5th Quintile	47.78	52.78	

† p values from Pearson chi-square test
