# Supplementary material for: Analysis of factors associated with extended recovery time after colonoscopy
Source: PLoS One. 2018 Jun 21;13(6):e0199246. doi: 10.1371/journal.pone.0199246 (PMC6013091; doi:10.1371/journal.pone.0199246)
Supplement: S2 Table — Results of multivariate logistic regression on the expanded variable set. (PDF) [file pone.0199246.s002.pdf]

Supplementary Table 2a: Multivariate Regression Results (Expanded Variable Set)

Variable	Odds Ratio of Long Recovery	Lower (95% CI)	Upper (95% CI)	p value
Gender: Male vs. Female	0.66	0.61	0.71	< 0.0001
Age*	1.08	1.05	1.11	< 0.0001
ASA Class:				
2 vs. 1	1.01	0.93	1.10	0.8211
3/4/Unknown vs. 1	1.57	1.22	2.00	0.0003
Year:				
2013 vs. 2012	0.83	0.73	0.94	0.0041
2014 vs. 2012	0.71	0.62	0.81	< 0.0001
2015 vs. 2012	0.54	0.45	0.64	< 0.0001
Endoscopist Quintile:				
2nd vs. 1st	1.23	1.10	1.37	0.0002
3rd vs. 1st	1.21	1.07	1.36	0.0023
4th vs. 1st	1.40	1.25	1.57	< 0.0001
5th vs. 1st	1.46	1.30	1.65	< 0.0001
Procedure RN Quintile:				
2nd vs. 1st	1.11	0.97	1.27	0.1322
3rd vs. 1st	1.09	0.96	1.25	0.1985
4th vs. 1st	1.16	1.02	1.32	0.0222
5th vs. 1st	1.27	1.11	1.45	0.0004
Recovery RN Quintile:				
2nd vs. 1st	1.75	1.51	2.03	< 0.0001
3rd vs. 1st	2.16	1.87	2.49	< 0.0001
4th vs. 1st	3.07	2.68	3.53	< 0.0001
5th vs. 1st	5.41	4.72	6.22	< 0.0001
Technician Quintile:				
2nd vs. 1st	1.03	0.87	1.23	0.7462
3rd vs. 1st	1.09	0.91	1.31	0.3581
4th vs. 1st	1.08	0.90	1.30	0.4028
5th vs. 1st	1.08	0.92	1.28	0.3488
Diphenhydramine*	1.19	1.15	1.24	< 0.0001
Fentanyl*	1.28	1.13	1.46	< 0.0001
Meperidine*	1.09	1.07	1.11	< 0.0001
Midazolam*	1.07	1.02	1.12	0.0030
Ondansetron*	1.16	1.12	1.20	< 0.0001

* Odds ratio per 10 year increase in age and increase in dosage of drugs: 10 mg for diphenhydramine and meperidine, 1 mg for midazolam and ondansetron, and 0.1 mg for fentanyl.

Supplementary Table 2b: Multivariate Regression Results (Expanded Variable Set)

Variable	Odds Ratio of Long Recovery	Lower (95% CI)	Upper (95% CI)	p value
Consciousness:				
Drowsy vs. Alert	1.71	1.56	1.88	< 0.0001
MSU*vs. Alert	2.22	1.75	2.81	< 0.0001
Unknown vs. Alert	2.04	0.90	4.21	0.0670
Preliminary Findings:				
Colitis vs. Normal Exam	1.49	1.19	1.86	0.0005
Diverticulosis vs. Normal Exam	1.03	0.92	1.14	0.6395
Hemorrhoids vs. Normal Exam	1.04	0.93	1.17	0.4772
Other/Unknown vs. Normal Exam	1.38	1.22	1.56	< 0.0001
Polyps vs. Normal Exam	1.04	0.94	1.16	0.4538
Poor Prep vs. Normal Exam	1.21	0.82	1.75	0.3345
Pain Level: >= 1/10 vs. 0/10	2.86	2.01	4.07	< 0.0001
Heart Rate Last Entry†	1.18	1.13	1.23	< 0.0001
Heart Rate Mean†	0.76	0.72	0.80	< 0.0001
Diastolic BP Standard Deviation†	1.43	1.30	1.57	< 0.0001
Mean BP Last Entry†	1.13	1.09	1.17	< 0.0001
Mean BP Mean†	0.72	0.69	0.76	< 0.0001

* MSU: Responds to mild or strong stimulation or unresponsive.

† Odds ratio per 10 bpm increase in heart rate and 10 mmHg increase in blood pressure
