# Supplementary material for: Analysis of factors associated with extended recovery time after colonoscopy
Source: PLoS One. 2018 Jun 21;13(6):e0199246. doi: 10.1371/journal.pone.0199246 (PMC6013091; doi:10.1371/journal.pone.0199246)
Supplement: S3 Table — Results of multivariate logistic regression models for each quintile of recovery room nurse. (PDF) [file pone.0199246.s003.pdf]

Supplementary Table 3a: Multivariate Regression Results (1st Recovery RN Quintile)

Variable	Odds Ratio of Long Recovery	Lower (95% CI)	Upper (95% CI)	p value
Consciousness:				
Drowsy vs. Alert	1.89	1.43	2.52	< 0.0001
MSU*vs. Alert	1.88	0.84	3.83	0.0992
Unknown vs. Alert	4.31	0.22	27.33	0.1875
Diastolic BP Last Entry†	0.78	0.67	0.92	0.0023
Diastolic BP Standard Deviation†	1.77	1.36	2.27	< 0.0001
Diastolic BP Mean†	0.88	0.63	1.23	0.4425
Diphenhydramine†	1.15	1.04	1.27	0.0044
Endoscopist Quintile:				
2nd vs. 1st	1.63	1.16	2.30	0.0053
3rd vs. 1st	1.74	1.22	2.49	0.0022
4th vs. 1st	1.50	1.06	2.14	0.0225
5th vs. 1st	1.69	1.17	2.46	5.3E-3
Gender: Male vs. Female	0.95	0.75	1.21	0.6871
Technician Quintile:				
2nd vs. 1st	0.93	0.58	1.51	0.7566
3rd vs. 1st	1.26	0.78	2.06	0.3492
4th vs. 1st	1.13	0.69	1.88	0.6258
5th vs. 1st	1.18	0.78	1.85	0.4462
Heart Rate Standard Deviation†	1.21	0.82	1.75	0.3238
IV Size:				
$\leq$ 20G vs. 22G	0.37	0.06	1.29	0.1873
24G vs. 22G	2.29	0.98	4.82	0.0394
Other/Unknown vs. 22G	3.23	0.72	10.34	0.0742
Mean BP Last Entry†	1.02	1.01	1.04	0.0027
Mean BP Mean†	0.99	0.96	1.01	0.2606
Mental Status: Other/Unknown vs. Alert	6.23	1.75	19.59	0.0025
Meperidine†	1.06	1.02	1.10	0.0070
Midazolam†	1.08	0.99	1.18	0.0892
Ondansetron†	1.36	1.25	1.48	< 0.0001
Pain Level: $\geq$ 1/10 vs. 0/10	6.92	2.9	15.58	< 0.0001
Planned Procedure:				
Diagnostic vs. Unspecified	0.8	0.56	1.13	0.2048
Screening vs. Unspecified	0.66	0.45	0.96	0.0324
Procedure Length†	1.01	1.01	1.02	0.0013
Procedure RN Quintile:				
2nd vs. 1st	1.01	0.71	1.44	0.9760
3rd vs. 1st	0.89	0.61	1.3	0.5492
4th vs. 1st	0.92	0.65	1.31	0.6410
5th vs. 1st	1.57	1.10	2.25	0.0143
Same as planned procedure:				
No/Unknown vs. Yes	2.38	1.02	5.09	0.0328
Peripheral Oxygen Saturation Last Entry†	1.04	1.01	1.09	0.0335
Year:				
2013 vs. 2012	0.87	0.59	1.3	0.4880
2014 vs. 2012	1.03	0.67	1.6	0.8763
2015 vs. 2012	0.85	0.49	1.49	0.5726

* MSU: Responds to mild or strong stimulation or unresponsive.

† Odds ratio per 10 year increase in age, 1 min increase in procedure length, 1 percentage point increase in oxygen saturation 10 bpm increase in heart rate, 10 mmHg increase in blood pressure and given increase in dosage of drugs: 10 mg for diphenhydramine and meperidine, 1 mg for midazolam and ondansetron, and 0.1 mg for fentanyl.

Supplementary Table 3b: Multivariate Regression Results (2nd Recovery RN Quintile)

Variable	Odds Ratio of Long Recovery	Lower (95% CI)	Upper (95% CI)	p value
Age†	1.08	1.00	1.18	0.0648
Consciousness (Alternate Scale):				
Arousable/Disoriented/Unresponsive vs. Awake/Oriented	3.22	1.28	7.34	0.0078
Consciousness:				
Drowsy vs. Alert	1.47	1.20	1.80	0.0002
MSU*vs. Alert	2.32	1.41	3.73	0.0007
Unknown vs. Alert	3.83	0.81	13.66	0.0534
Diastolic BP Last Entry†	0.79	0.70	0.90	0.0003
Diastolic BP Standard Deviation†	1.14	0.82	1.58	0.4421
Diastolic BP Mean†	1.55	1.11	2.17	0.0097
Diphenhydramine†	1.15	1.06	1.25	0.0008
Endoscopist Quintile:				
2nd vs. 1st	1.15	0.90	1.47	0.2621
3rd vs. 1st	1.21	0.92	1.59	0.1709
4th vs. 1st	1.44	1.13	1.84	0.0035
5th vs. 1st	1.31	1.01	1.71	0.0044
Preliminary Findings:				
Colitis vs. Normal Exam	1.82	1.16	2.82	0.0080
Diverticulosis vs. Normal Exam	1.13	0.89	1.44	0.3074
Hemorrhoids vs. Normal Exam	1.02	0.79	1.33	0.8696
Other/Unknown vs. Normal Exam	1.65	1.27	2.13	0.0002
Polyps vs. Normal Exam	1.02	0.80	1.29	0.8838
Poor Prep vs. Normal Exam	1.20	0.47	2.65	0.6736
Gender: Male vs. Female	0.68	0.56	0.82	< 0.0001
Technician Quintile:				
2nd vs. 1st	0.81	0.55	1.20	0.2897
3rd vs. 1st	0.94	0.64	1.42	0.7773
4th vs. 1st	1.09	0.73	1.65	0.6616
5th vs. 1st	1.09	0.77	1.59	0.6290
Heart Rate Standard Deviation†	1.43	1.11	1.84	0.0058
Mean BP Last Entry†	1.12	0.99	1.27	0.0752
Mean BP Mean†	0.57	0.36	0.91	0.0186
Mean BP Standard Deviation†	1.07	0.77	1.47	0.6758
Meperidine†	1.06	1.03	1.10	0.0001
Midazolam†	1.16	1.09	1.25	< 0.0001
Ondansetron†	1.15	1.07	1.24	0.0002
Pain Level: $\geq 1/10$ vs. 0/10	6.54	3.23	13.31	< 0.0001
Positioning aids:				
Bath Blankets vs. Other/None	0.68	0.47	0.99	0.0429
Pillows vs. Other/None	0.90	0.69	1.19	0.4588
Procedure RN Quintile:				
2nd vs. 1st	0.89	0.67	1.18	0.4074
3rd vs. 1st	0.85	0.64	1.13	0.2631
4th vs. 1st	0.96	0.74	1.26	0.7928
5th vs. 1st	1.06	0.81	1.41	0.6585
Respiration Rate Last Entry	1.01	1.00	1.03	0.0683
Respirations: Other/Unknown vs. Unlabored	2.46	1.00	5.59	0.0382
Rhythm Last Entry:				
AF/AFL vs. NSR	3.73	2.03	6.57	< 0.0001
Other/Unknown vs. NSR	1.30	0.70	2.27	0.3831
Regular vs. NSR	1.41	1.07	1.85	0.0135
SB vs. NSR	1.21	0.93	1.55	0.1481
ST vs. NSR	1.40	0.60	3.04	0.4096
Peripheral Oxygen Saturation Mean†	0.98	0.93	1.04	0.5197
Peripheral Oxygen Saturation Standard Deviation†	1.03	0.97	1.09	0.2943
Systolic BP Last Entry†	1.14	1.05	1.25	0.0029
Systolic BP Mean†	0.90	0.72	1.11	0.3169
Systolic BP Standard Deviation†	1.11	0.88	1.40	0.3701
Year:				
2013 vs. 2012	0.71	0.53	0.96	0.0247
2014 vs. 2012	0.64	0.46	0.89	0.0069
2015 vs. 2012	0.47	0.32	0.70	0.0002

* MSU: Responds to mild or strong stimulation or unresponsive.

† Odds ratio per 10 year increase in age, 1 min increase in procedure length, 1 percentage point increase in oxygen saturation 10 bpm increase in heart rate, 10 mmHg increase in blood pressure and given increase in dosage of drugs: 10 mg for diphenhydramine and meperidine, 1 mg for midazolam and ondansetron, and 0.1 mg for fentanyl.

Supplementary Table 3c: Multivariate Regression Results (3rd Recovery RN Quintile)

Variable	Odds Ratio of Long Recovery	Lower (95% CI)	Upper (95% CI)	p value
Consciousness:				
Drowsy vs. Alert	2.01	1.66	2.44	< 0.0001
MSU*vs. Alert	3.22	2.03	5.03	< 0.0001
Unknown vs. Alert	1.75	0.26	7.09	0.4857
Diastolic BP Last Entry†	0.89	0.8	0.98	0.0176
Diastolic BP Standard Deviation†	1.79	1.5	2.13	< 0.0001
Diastolic BP Mean†	0.96	0.78	1.18	0.6991
Diphenhydramine†	1.20	1.12	1.30	< 0.0001
Endoscopist Quintile:				
2nd vs. 1st	1.36	1.10	1.68	0.0039
3rd vs. 1st	1.42	1.12	1.79	0.0031
4th vs. 1st	1.54	1.24	1.92	0.0001
5th vs. 1st	1.50	1.20	1.89	0.0004
Gender: Male vs. Female	0.67	0.57	0.77	< 0.0001
Technician Quintile:				
2nd vs. 1st	0.92	0.66	1.28	0.5985
3rd vs. 1st	1.02	0.73	1.45	0.8929
4th vs. 1st	0.99	0.70	1.40	0.9458
5th vs. 1st	1.09	0.80	1.49	0.5928
Heart Rate Mean†	0.87	0.82	0.94	0.0002
Mean BP Last Entry†	1.28	1.18	1.40	< 0.0001
Mean BP Mean†	0.74	0.62	0.87	0.0004
Meperidine†	1.03	1.00	1.06	0.0231
Ondansetron†	1.14	1.07	1.21	0.0001
Pain Level: $\geq$ 1/10 vs. 0/10	3.30	1.61	6.56	0.0008
Procedure RN Quintile:				
2nd vs. 1st	1.23	0.96	1.6	0.1092
3rd vs. 1st	1.2	0.93	1.55	0.1752
4th vs. 1st	1.33	1.05	1.71	0.0209
5th vs. 1st	1.25	0.96	1.62	0.0957
Respiration Rate Standard Deviation	1.04	1.01	1.07	0.0155
Total Number of Polyps	0.97	0.92	1.02	0.2540
Year:				
2013 vs. 2012	0.74	0.57	0.95	0.0202
2014 vs. 2012	0.65	0.50	0.86	0.0023
2015 vs. 2012	0.57	0.41	0.79	0.0008

* MSU: Responds to mild or strong stimulation or unresponsive.

† Odds ratio per 10 year increase in age, 1 min increase in procedure length, 1 percentage point increase in oxygen saturation 10 bpm increase in heart rate, 10 mmHg increase in blood pressure and given increase in dosage of drugs: 10 mg for diphenhydramine and meperidine, 1 mg for midazolam and ondansetron, and 0.1 mg for fentanyl.

Supplementary Table 3d: Multivariate Regression Results (4th Recovery RN Quintile)

Variable	Odds Ratio of Long Recovery	Lower (95% CI)	Upper (95% CI)	p value
Consciousness:				
Drowsy vs. Alert	1.58	1.36	1.84	< 0.0001
MSU*vs. Alert	2.42	1.64	3.55	< 0.0001
Unknown vs. Alert	1.30	0.29	4.27	0.6976
Diastolic BP Last Entry†	0.91	0.84	1.00	0.0439
Diastolic BP Standard Deviation†	1.39	1.19	1.62	< 0.0001
Diastolic BP Mean†	1.03	0.84	1.26	0.7722
Diphenhydramine†	1.18	1.10	1.26	< 0.0001
Endoscopist Quintile:				
2nd vs. 1st	1.16	0.97	1.38	0.1049
3rd vs. 1st	1.02	0.84	1.25	0.8207
4th vs. 1st	1.15	0.95	1.39	0.1411
5th vs. 1st	1.35	1.11	1.63	0.0021
Fentanyl†	1.11	0.90	1.37	0.3417
Preliminary Findings:				
Colitis vs. Normal Exam	1.29	0.89	1.85	0.1717
Diverticulosis vs. Normal Exam	1.01	0.85	1.20	0.9308
Hemorrhoids vs. Normal Exam	1.00	0.82	1.20	0.9771
Other/Unknown vs. Normal Exam	1.12	0.91	1.39	0.2851
Polyps vs. Normal Exam	0.86	0.72	1.02	0.0810
Poor Prep vs. Normal Exam	1.82	1.00	3.18	0.0415
Gender: Male vs. Female	0.72	0.63	0.82	< 0.0001
Heart Rate Mean†	0.86	0.81	0.92	< 0.0001
Mean BP Last Entry†	1.09	1.00	1.18	0.0432
Mean BP Mean†	0.67	0.57	0.80	< 0.0001
Mental Status: Other/Unknown vs. Alert	4.14	1.50	11.27	0.0050
Meperidine†	1.07	1.04	1.11	0.0001
Midazolam†	1.08	1.00	1.16	0.0466
O_2 Last Entry:				
2 L/min vs. Other/Unknown	1.34	0.59	3.35	0.5088
Room air vs. Other/Unknown	1.78	0.87	4.13	0.1401
Ondansetron†	1.15	1.08	1.23	< 0.0001
Pain Level: $\geq 1/10$ vs. 0/10	2.01	1.04	3.77	0.0323
Planned Procedure:				
Diagnostic vs. Unspecified	0.88	0.69	1.11	0.2830
Screening vs. Unspecified	0.77	0.60	0.99	0.0390
Procedure RN Quintile:				
2nd vs. 1st	1.23	0.98	1.55	0.0752
3rd vs. 1st	1.27	1.01	1.60	0.0381
4th vs. 1st	1.08	0.87	1.35	0.4669
5th vs. 1st	1.19	0.95	1.49	0.1413
Scheduled Procedure Room	1.01	0.99	1.02	0.2049
Peripheral Oxygen Saturation Last Entry†	1.01	1.00	1.02	0.1709
Systolic BP Last Entry†	1.12	1.06	1.19	0.0002
Year:				
2013 vs. 2012	0.92	0.75	1.13	0.4136
2014 vs. 2012	0.81	0.64	1.02	0.0692
2015 vs. 2012	0.70	0.50	0.98	0.0370

* MSU: Responds to mild or strong stimulation or unresponsive.

† Odds ratio per 10 year increase in age, 1 min increase in procedure length, 1 percentage point increase in oxygen saturation 10 bpm increase in heart rate, 10 mmHg increase in blood pressure and given increase in dosage of drugs: 10 mg for diphenhydramine and meperidine, 1 mg for midazolam and ondansetron, and 0.1 mg for fentanyl.

Supplementary Table 3e: Multivariate Regression Results (5th Recovery RN Quintile)

Variable	Odds Ratio of Long Recovery	Lower (95% CI)	Upper (95% CI)	p value
Consciousness:				
Drowsy vs. Alert	1.83	1.58	2.12	< 0.0001
MSU*vs. Alert	2.11	1.40	3.16	0.0003
Unknown vs. Alert	3.33	0.94	11.02	0.0493
Diphenhydramine†	1.13	1.05	1.21	0.0013
Endoscopist Quintile:				
2nd vs. 1st	1.35	1.13	1.61	0.0008
3rd vs. 1st	1.39	1.15	1.69	0.0008
4th vs. 1st	1.40	1.17	1.68	0.0003
5th vs. 1st	1.46	1.19	1.79	0.0003
Fentanyl†	1.14	0.93	1.41	0.2093
Preliminary Findings:				
Colitis vs. Normal Exam	1.30	0.89	1.87	0.1689
Diverticulosis vs. Normal Exam	1.13	0.95	1.35	0.1535
Hemorrhoids vs. Normal Exam	1.01	0.83	1.22	0.9489
Other/Unknown vs. Normal Exam	1.50	1.21	1.86	0.0002
Polyps vs. Normal Exam	1.23	1.04	1.46	0.0175
Poor Prep vs. Normal Exam	1.00	0.53	1.83	0.9905
Gender: Male vs. Female	0.61	0.54	0.69	< 0.0001
Technician Quintile:				
2nd vs. 1st	1.34	0.98	1.85	0.0667
3rd vs. 1st	1.27	0.91	1.78	0.1652
4th vs. 1st	1.23	0.89	1.72	0.2101
5th vs. 1st	1.15	0.85	1.56	0.3814
Heart Rate Mean†	0.87	0.82	0.93	< 0.0001
Heart Rate Standard Deviation†	1.09	0.89	1.34	0.3759
Lidocaine Spray	1.12	0.94	1.33	0.2188
Mean BP Last Entry†	1.13	1.04	1.22	0.0025
Mean BP Mean†	0.82	0.7	0.97	0.0186
Mean BP Standard Deviation†	1.14	0.96	1.34	0.1233
Meperidine†	1.06	1.02	1.10	0.0011
Midazolam†	1.04	0.97	1.12	0.2946
O_2 Last Entry:				
2 L/min vs. Other/Unknown	1.04	0.45	2.60	0.9295
Room air vs. Other/Unknown	1.53	0.74	3.50	0.2789
Ondansetron†	1.14	1.07	1.22	< 0.0001
Pain Level: $\geq 1/10$ vs. 0/10	1.53	0.83	2.77	0.1644
Planned Procedure:				
Diagnostic vs. Unspecified	0.77	0.61	0.98	0.0344
Screening vs. Unspecified	0.60	0.46	0.77	< 0.0001
Procedure RN Quintile:				
2nd vs. 1st	1.01	0.81	1.26	0.9352
3rd vs. 1st	0.89	0.71	1.11	0.2900
4th vs. 1st	1.11	0.90	1.36	0.3458
5th vs. 1st	1.15	0.92	1.43	0.2249
Systolic BP Last Entry†	1.06	0.99	1.13	0.0819
Systolic BP Mean†	0.93	0.82	1.04	0.2065
Systolic BP Standard Deviation†	1.17	0.99	1.37	0.0626
Year:				
2013 vs. 2012	0.84	0.68	1.05	0.1246
2014 vs. 2012	0.73	0.57	0.93	0.0113
2015 vs. 2012	0.70	0.49	1.00	0.0483

* MSU: Responds to mild or strong stimulation or unresponsive.

† Odds ratio per 10 year increase in age, 10 bpm increase in heart rate, 10 mmHg increase in blood pressure and given increase in dosage of drugs: 10 mg for diphenhydramine and meperidine, 1 mg for midazolam and ondansetron, and 0.1 mg for fentanyl.
