# Supplementary figures and images for: Analysis of factors associated with extended recovery time after colonoscopy
Source: PLoS One. 2018 Jun 21;13(6):e0199246. doi: 10.1371/journal.pone.0199246 (PMC6013091; doi:10.1371/journal.pone.0199246)

# Recovery Time Averaged By Hospital Staff

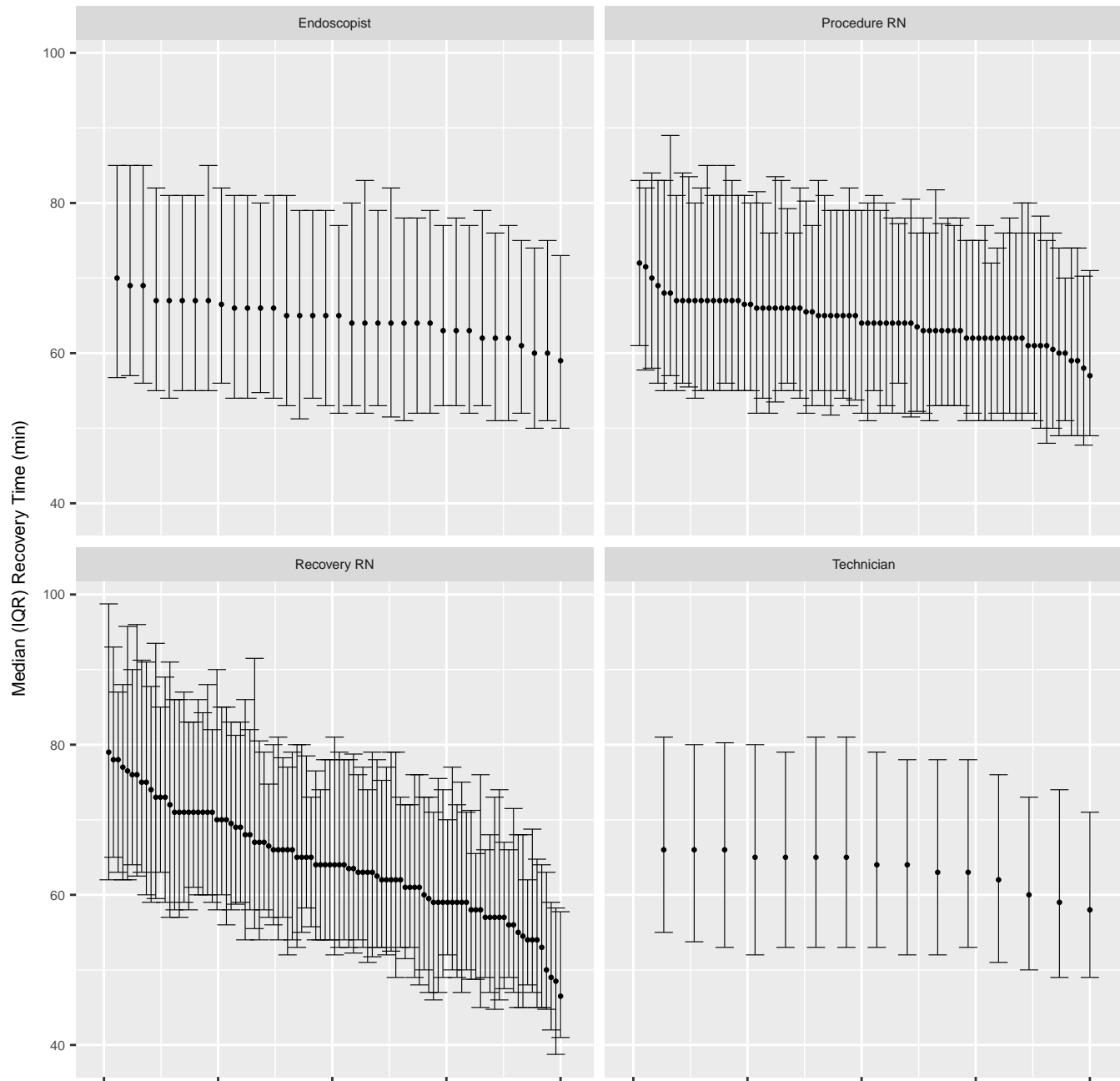

Supplement: S1 Fig — Median recovery time by hospital personnel with interquartile range. Each point represents one individual or the aggregated data of individuals involved in a small number of procedures, as described in the methods section. (PDF) [file pone.0199246.s004.pdf]
