# Supplementary material for: Analysis of factors associated with extended recovery time after colonoscopy
Source: PLoS One. 2018 Jun 21;13(6):e0199246. doi: 10.1371/journal.pone.0199246 (PMC6013091; doi:10.1371/journal.pone.0199246)
Supplement: S2 Fig — Percentage of procedures with recovery time greater than 85 minutes by hospital personnel with Wilson confidence interval. Each point represents one individual or the aggregated data of individuals involved in a small number of procedures, as described in the methods section. (PDF) [file pone.0199246.s005.pdf]

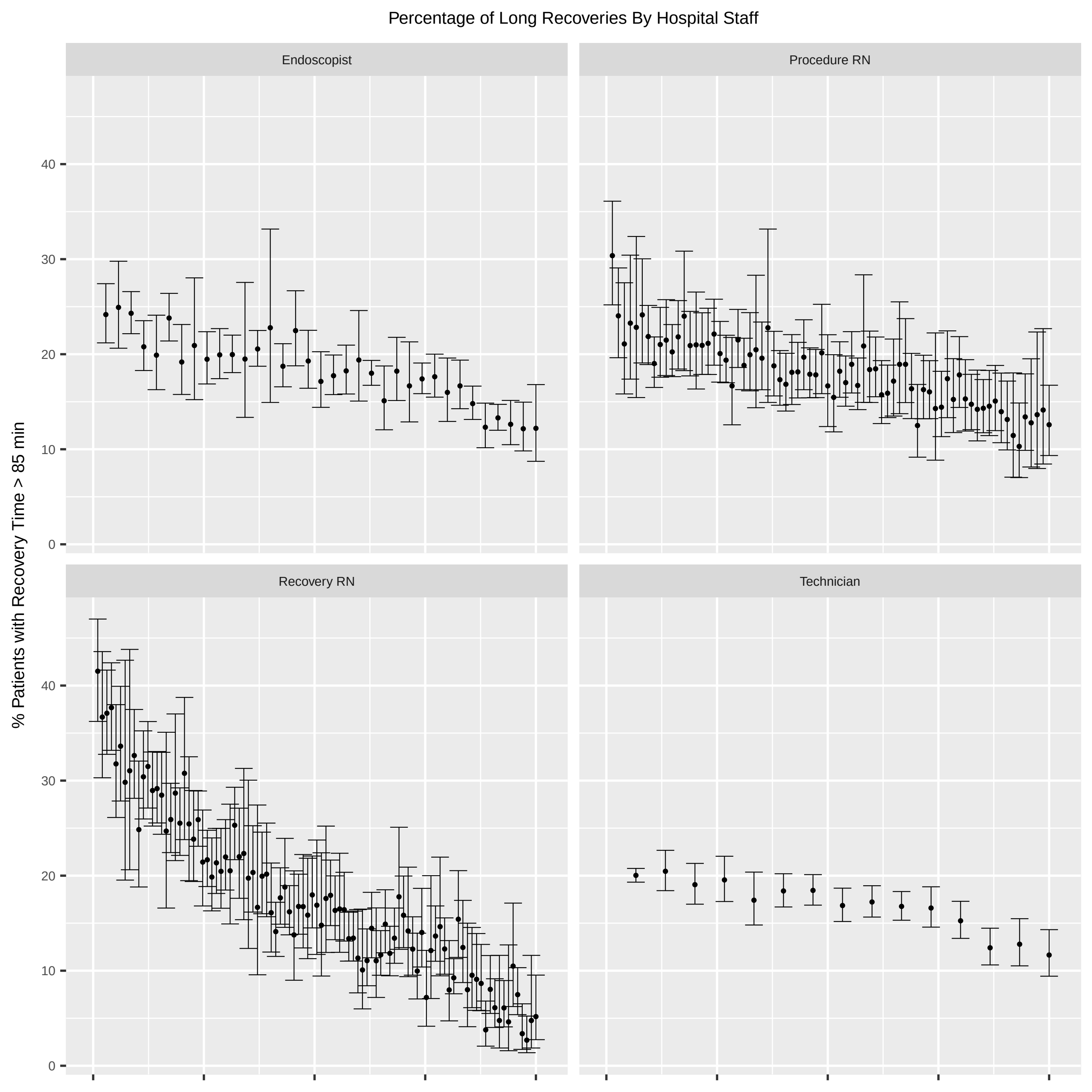

Percentage of Long Recoveries By Hospital Staff
Endoscopist
Procedure RN
Recovery RN
Technician
% Patients with Recovery Time > 85 min
0
10
20
30
40
